# Ranking and clustering G20 healthcare systems: a framework for U.S. reform

**DOI:** 10.3389/fpubh.2026.1810123

**Published:** 2026-04-10

**Authors:** Eliasz Powzun-Palczuk, Dariusz Walkowiak

**Affiliations:** 1 Sibilla Solutions, Nicosia, Cyprus; 2 Department of Organization and Management in Health Care, Uniwersytet Medyczny im Karola Marcinkowskiego w Poznaniu, Poznań, Poland

**Keywords:** cluster analysis, comparative health policy, G20 health systems, health infrastructure, health outcomes, healthcare system performance, healthcare workforce, U.S. healthcare reform

## Abstract

**Objectives:**

To evaluate and compare healthcare system performance across G20 countries and Poland using multidimensional indicators of health outcomes, infrastructure, workforce capacity, and disease burden. The study examines structural patterns associated with high performance and implications for U. S. health system reform.

**Methods:**

We analysed 34 standardised indicators from WHO, OECD, and national databases. Z-score normalisation enabled cross-country comparability, and hierarchical cluster analysis using Ward’s method validated performance groupings. Countries were classified into five tiers.

**Results:**

Healthcare performance varied substantially across countries. Higher GDP per capita (PPP) was associated with better outcomes, though outliers showed that financial resources alone are insufficient. Japan, South Korea, and Australia formed the highest-performing group, characterised by coherent financing, strong regulation, and preventive investment. The United States, despite the highest spending, underperformed relative to most high-income systems.

**Conclusion:**

Economic capacity facilitates but does not guarantee strong health system performance. The countries in the highest-performing group share several institutional characteristics, including regulated insurance markets, structured cost-sharing mechanisms, and strong primary care systems. The U.S. may benefit from adopting elements of regulated, solidarity-based multi-payer models observed in higher-performing countries.

## Introduction

Healthcare systems are central to social and economic resilience, shaping not only population health but also labour productivity, social cohesion, and governments’ ability to meet Sustainable Development Goals. In an era of demographic ageing, rising chronic disease burden, and fiscal pressure, understanding why some systems achieve better results than others is increasingly important. Cross-country comparisons provide a way to move beyond anecdote and ideology, offering structured evidence on what works, for whom, and under what conditions (1–4).

Healthcare systems across the world differ substantially in their institutional architecture, policy philosophy, and governance arrangements. Despite these differences, most systems face a set of common structural pressures, including population ageing, rising costs associated with technological progress, and persistent inequalities in access to care. As a result, comparative research increasingly focuses on identifying structural characteristics that allow certain systems to achieve better outcomes or higher efficiency under similar economic constraints (3, 5–9).

Healthcare systems differ markedly across nations, shaped by variations in economic development, public health priorities, governance structures, and historical legacies. Comparative evaluation of these systems enables policymakers to identify best practices, detect inefficiencies, and guide evidence-based reform (10). International frameworks - such as those developed by the World Health Organisation (WHO), the Organisation for Economic Co-operation and Development (OECD), and Bloomberg- emphasise multidimensional assessment approaches that incorporate indicators such as workforce availability, infrastructure, health outcomes, and disease burden.

Comparative health policy literature often classifies healthcare systems according to their financing and governance structures. One commonly used typology distinguishes between the Beveridge model- funded primarily through general taxation and characterised by strong state regulation and universal coverage- and the Bismarck model, which relies on social health insurance contributions shared between employers and employees and administered through multiple regulated insurance funds. In practice, many contemporary healthcare systems combine elements of both models, creating hybrid institutional arrangements that balance solidarity-based financing with regulated competition among insurers and providers (11–14).

Over the past two decades, these comparative efforts have generated a range of indices and scorecards, yet they often differ in scope, methodology, and emphasis. Some rankings focus predominantly on outcomes or expenditure; others privilege financial protection or efficiency. As a result, policymakers are frequently confronted with fragmented signals that are difficult to reconcile. There is a need for integrated, transparent approaches that combine multiple dimensions of performance while remaining interpretable for decision-makers.

Most modern comparative frameworks evaluate healthcare systems across multiple performance domains. For example, international benchmarking initiatives such as those conducted by the Commonwealth Fund assess systems across dimensions including access to care, care processes (such as preventive services and coordination), administrative efficiency, equity in access and outcomes, and population health outcomes such as life expectancy and avoidable mortality. These multidimensional approaches reflect the growing recognition that health system performance cannot be adequately captured by a single indicator (6, 13, 15–17).

Several influential comparative studies have attempted to evaluate health system performance across countries. The WHO World Health Report 2000 introduced one of the earliest multidimensional frameworks for assessing health system performance at a global scale, combining outcome, responsiveness, and financing indicators (18). Subsequent comparative analyses- including those conducted by the Commonwealth Fund and OECD- have continued to benchmark health systems across high-income countries, often emphasising outcome measures, financial protection, and system efficiency (2, 3, 5, 19). However, these approaches frequently rely on predefined weighting schemes or focus primarily on specific performance dimensions. The present study contributes to this literature by applying a transparent *z*-score standardisation framework combined with hierarchical clustering to identify structural performance tiers across major economies.

Building on these frameworks, this study evaluates and ranks the healthcare systems of the G20 countries alongside Poland. The G20 comprises the world’s largest economies, each exemplifying unique models of healthcare delivery. Poland, currently ranked as the 21st largest economy, was included as a representative of a rapidly developing EU member state with a transitional health system that evolved from a centrally planned structure. Although Poland is not a formal member of the G20, it has recently been invited by the United States presidency to participate in selected meetings as a guest country, reflecting its growing economic and geopolitical relevance. Its inclusion provides relevant insights into performance in post-socialist and middle-income contexts.

Beyond constructing a cross-national ranking, the study explores how key structural characteristics relate to outcomes across different performance tiers. A specific focus is placed on the United States, where exceptionally high healthcare spending has failed to yield commensurate results. By benchmarking against higher-performing systems, the study proposes a reform-oriented framework that may inform future policy development. The primary aim is to assess healthcare system effectiveness using standardised statistical indicators and to examine how structural differences relate to outcomes. This includes an analysis of how classic system typologies- such as the Beveridge, Bismarck, and Semashko models described by Roemer (20) - continue to shape access, financing, and governance structures in different contexts, with Poland serving as a valuable comparative case for transitional systems.

In doing so, the study seeks to bridge three gaps in the existing literature: the lack of a unified ranking that covers G20 countries using a broad indicator set; limited use of cluster analysis to validate performance groupings; and insufficient translation of comparative findings into concrete reform options for high-spending but underperforming systems such as that of the United States. The analysis therefore aims not only to describe variation but also to inform practical debates on how different countries can move toward more equitable, efficient, and resilient healthcare systems.

## Methods

This study used a cross-sectional ecological design, with the country as the unit of analysis. The primary objective was to compare overall health system performance across a set of large economies using a consistent set of indicators and a transparent standardisation procedure. The analytic strategy was structured to balance statistical rigour with interpretability for policymakers, emphasising methods that can be replicated in future comparative work.

Rather than constructing a single weighted index of healthcare system performance, the analytical strategy focuses on identifying structural similarities and differences between national systems using multivariate statistical techniques. This approach allows patterns in the data to emerge empirically while avoiding the need to impose subjective weights on individual indicators (2, 3, 5, 21, 22).

The analysis draws on 34 statistical indicators grouped into five domains: availability of healthcare personnel, infrastructure capacity, population health outcomes, infectious disease prevalence, and frequency of cesarean sections. Countries analysed include the G20 members, totalling 20 health systems. Data were sourced primarily from the WHO’s Global Health Observatory (23), complemented by national statistical agencies where necessary. Most indicators refer to the most recent available observations between 2019 and 2023, reflecting typical reporting delays in international health statistics. To ensure valid cross-country comparison, all data points were standardised using the *z*-score normalisation method. This method accounts for differences in scale and units of measurement, allowing for integration of heterogeneous indicators into a single composite framework. The z-score transformation was used solely to standardise variables measured on different scales and units, enabling meaningful multivariate comparison across indicators. This transformation does not in itself constitute a composite index but serves as a preparatory step for multivariate analysis.

For indicators where a higher value indicates better performance (e.g., life expectancy), the original z-score was retained. For indicators where lower values are preferable (e.g., infant mortality), z-scores were inverted to preserve consistent interpretation across metrics. The selected indicators reflect core dimensions of healthcare performance and are consistent with criteria used in WHO’s World Health Statistics (2023) (24), the OECD’s Health at a Glance (2023) (25), and the Global Burden of Disease (GBD) study from the Institute for Health Metrics and Evaluation (IHME) (26). These domains reflect the conceptual framework introduced by Murray and Frenk for assessing system performance through responsiveness, fairness, and outcome metrics (27). Indicators include key health outcome metrics such as physicians and nurses per capita, hospital bed density, maternal and infant mortality, healthy life expectancy at different ages, and prevalence of infectious diseases like HIV, polio, and diphtheria.

To complement the ranking, a hierarchical cluster analysis was conducted to explore underlying patterns among countries. This analysis used Ward’s minimum variance method and Euclidean distance as the similarity measure, grouping countries based on their 34 normalised healthcare indicators. Data processing and visualisation were performed in Python 3.11 using the scipy.cluster.hierarchy module. Cluster analysis is widely used in comparative health system research because it allows countries with broadly similar demographic and institutional characteristics to be analysed together. This approach helps avoid misleading comparisons between systems operating under very different economic or epidemiological conditions. Countries were subsequently categorised into five performance-based groups using their aggregated *z*-scores. *I*n parallel, hierarchical cluster analysis was conducted using the full set of standardised indicators to explore natural groupings among countries in the multidimensional indicator space. Unlike simple rankings, clustering identifies groups of countries that share similar structural and outcome profiles across multiple variables simultaneously. Cluster analysis validated these groupings, confirming natural divisions based on shared structural and outcome profiles. These clusters served as the foundation for the cross-country comparison in the results and policy interpretation sections. This two-step method - ranking by standardised score and validation through clustering - ensures both statistical robustness and meaningful interpretability of healthcare system performance across diverse national contexts.

In line with the study objective, no attempt was made to weight individual indicators according to predefined policy priorities; instead, all variables contributed equally to the composite score. This equal-weighting approach was chosen to preserve methodological transparency and avoid introducing normative assumptions regarding the relative importance of specific health system dimensions. While alternative weighting schemes could be applied, they typically require subjective judgments or optimisation procedures that may reduce interpretability in comparative policy analysis. The resulting tiers and clusters should therefore be interpreted as multidimensional performance profiles rather than definitive judgments about any single aspect of a country’s health system. This choice favours transparency and reproducibility in cross-country comparative research.

## Results

Overall, the descriptive analysis and standardised scoring revealed substantial heterogeneity in healthcare system performance across the 20 countries examined. Marked variation was observed not only between income groups but also within similar economic clusters, underscoring the complex role of policy design, system structure, and resource allocation in shaping health outcomes. The combination of z-score aggregation and hierarchical clustering allowed both linear and non-linear patterns to emerge, providing a comprehensive picture of cross-country similarities and divergences.

The analysis revealed a strong correlation between a country’s level of economic development, measured by GDP per capita (PPP), and overall healthcare system performance. Importantly, GDP per capita was not used as an input variable in the clustering procedure; rather, it emerged as a contextual predictor when interpreting the composite performance scores derived from the 34 standardised health system indicators. Based on aggregated *z*-scores derived from 34 standardised indicators, countries were grouped into five performance-based tiers, each characterised by specific structural, financial, and outcome-related patterns. Please, see Supplementary Appendix 1. After performing the z-score analysis, the countries were grouped into five distinct performance tiers:

Group 1: Japan, South Korea, Australia.
Group 2: France, Italy, Canada, Germany, United Kingdom.
Group 3: USA, Poland, China, Turkey, Saudi Arabia, Argentina.
Group 4: Russia, Mexico, Brazil.
Group 5: Indonesia, India, South Africa.

### Cross-cutting findings and cluster validation

Across all groups, economic development emerged as a critical predictor of healthcare performance. The analysis suggests a threshold of ~$40,000 GDP per capita (PPP) as the tipping point for high-functioning systems. However, outliers like Russia (high GDP, low health outcomes) and China (moderate GDP, rising performance) underscore that efficiency and equity depend on more than wealth alone.

**Figure 1:**
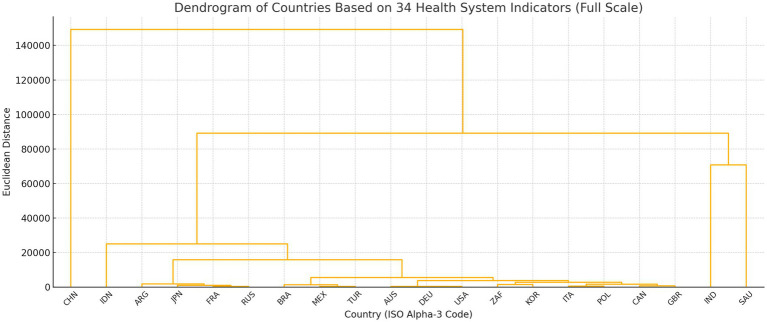
Cluster analysis results (scale: 140′000).

**Figure 2:**
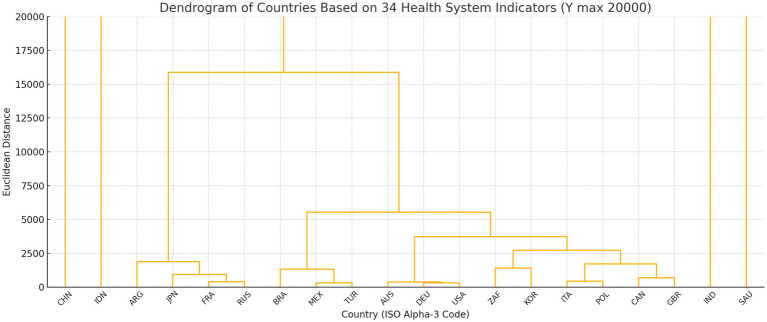
Cluster analysis results (scale: 20′000).

To validate the robustness of the performance tiers derived from the composite z-score analysis, a supplementary hierarchical cluster analysis was conducted using Ward’s minimum variance method and Euclidean distance on the full matrix of 34 standardised healthcare indicators. Importantly, GDP per capita was not included as an input variable in the clustering procedure; rather, clustering was performed exclusively on the healthcare system indicators themselves. Macroeconomic indicators such as GDP per capita were intentionally excluded from the clustering variables in order to avoid conflating health system performance with overall economic development. Economic capacity was therefore analysed only during the interpretation stage. The results showed that 16 out of 20 countries clustered into a single primary group, while China, India, Indonesia, and Saudi Arabia formed separate clusters, indicating divergent structural profiles (see Figures 1, 2). These findings support the robustness of the performance tiers and provide a basis for targeted policy analysis and benchmarking, particularly for countries such as the United States, where high spending does not yield proportional outcomes.

## Discussion

The findings of this comparative analysis highlight both predictable and unexpected performance patterns across the G20 and Poland. While economic development remains a foundational determinant of healthcare system strength, structural, institutional, and behavioural factors substantially modify outcomes. These results illustrate that financial capacity alone does not guarantee efficiency or equity. Instead, system architecture- particularly the balance between public financing, private provision, and regulatory oversight-emerges as a major driver of cross-national differences. The Discussion contextualises the performance of each country group, identifies recurring structural traits among high performers, and links these observations to potential pathways for healthcare reform in the United States.

Global comparisons also reveal substantial variation in healthcare expenditure across countries. The United States represents the most prominent outlier: it spends a substantially larger share of GDP on healthcare than other high-income economies, and per-capita spending is more than double that of many peer nations. Evidence suggests that these higher costs are driven primarily by higher prices for healthcare services, pharmaceuticals, and administrative complexity rather than significantly higher utilisation of services (5, 15, 17, 22, 28).

Beyond economic capacity, the top-performing countries share several structural advantages: relatively low administrative fragmentation, stable long-term financing mechanisms, strong gatekeeping and referral systems, and investments in preventive care and early detection. These features enable efficient allocation of resources, better continuity of care, and broad access to essential services. Importantly, these strengths persist across different institutional models- Bismarckian, Beveridge-type, and hybrid- suggesting that design coherence matters more than adherence to a specific historical template.

The results confirm a strong correlation between economic development and healthcare system performance. Countries with a GDP per capita (PPP) above ~$40,000 tend to achieve better health outcomes, more robust infrastructure, and overall stronger system performance. These findings are consistent with prior comparative work, including WHO’s World Health Reports and OECD’s Health at a Glance, which highlight the role of economic capacity in enabling care access, infrastructure investment, and health equity (24, 25). This study extends these approaches by integrating a broad indicator set and validating performance tiers through cluster analysis. While institutional models vary, top-performing systems often combine public provision with regulated cost-sharing or supplementary private coverage, balancing sustainability and access (7).

Efficiency comparisons often distinguish between healthcare system inputs- such as financial resources, workforce capacity, and infrastructure- and outputs, including service volumes and population health outcomes. Previous research suggests that a significant share of global healthcare expenditure may be lost due to systemic inefficiencies, including administrative complexity, duplication of services, and poorly coordinated care (6, 7, 17, 29, 30).

Group 1 countries scored above +25 points, combining strong outcomes, efficient cost-sharing, and comprehensive infrastructure. All three- Japan, South Korea, and Australia- are Asia-Pacific nations employing hybrid models with public oversight and market incentives. In Japan, healthcare costs are split 30/70 between patients and insurers. Contributions are equally divided between employers and employees (10–13%), and patients access specialists directly. Strong regulation ensures transparency and controls inflation, contributing to world-leading life expectancy and equity (31). South Korea uses a similar structure with universal mandatory insurance, with subsidies or free care for low-income groups (32). Australia blends public funding with near-universal private coverage: citizens pay a 2% Medicare levy, and private insurance is encouraged via tax penalties for higher earners without coverage (33).

A cross-cutting characteristic of Group 1 systems is their ability to integrate public financing with market incentives without compromising equity. These systems maintain comparatively low administrative overhead and consistent national regulation, limiting regional variation in service quality. Cultural and behavioural factors- such as high health literacy in Japan and South Korea- may further reduce preventable disease burden. This combination helps explain high outcomes despite spending a smaller share of GDP on health than the United States.

Group 2 includes advanced economies with robust systems and a shared commitment to universality- via the Beveridge model (UK, Italy, Canada), Bismarck model (Germany), or hybrids (France). All feature high GDP per capita and broad access to essential services. Beveridge systems are tax-funded and provide free care at the point of use, though long wait times have fostered private markets. Germany’s Bismarck model uses statutory health insurance with equal employer-employee contributions (14.6%). France employs a 70/30 cost-sharing model, supported by private insurance (34, 35). Despite structural differences, these countries converge in outcomes- particularly longevity and maternal/infant health. Minor inefficiencies, such as wait times and regional gaps, slightly lower their scores.

Although Group 2 countries differ in institutional design, they converge on core operational elements: broad population coverage, stable funding, and statutory guarantees of essential services. Their challenges- especially wait times in Beveridge systems and rising costs in social insurance models- reflect growing fiscal pressure but do not undermine core performance metrics. These systems illustrate that universality can coexist with financial sustainability when supported by strong primary care networks and regulated pricing.

Group 3 spans both developed and developing economies, with scores from −5 to +6. The U. S. ranks surprisingly low despite spending over 17% of GDP on healthcare. High uninsured rates, fragmented coverage, and weak price regulation contribute to inefficiency, while profit-driven incentives and pricing opacity further reduce performance. Poland has a single-payer model based on compulsory insurance. Structurally solid but underfunded (9% vs. Germany’s 14.6%), it struggles with access disparities and equipment shortages. China combines public insurance with mandatory private coverage for foreigners; multiple schemes- such as Urban Employee Basic Medical Insurance and rural cooperatives- support broad coverage, though benefit gaps and regional inequality persist (36). Turkey follows a model similar to Poland’s, but with lower funding. In Saudi Arabia, citizens receive free care, while expatriates- about one-third of the population- must use private insurance, creating a dual-track system (37). Argentina guarantees universal care constitutionally, but uneven service quality and staff shortages- especially outside urban areas- reduce effectiveness; the private sector often compensates for these gaps (38).

Group 3 provides the clearest illustration of divergence between spending and outcomes. The U. S. and Saudi Arabia achieve only moderate performance despite high expenditure, underscoring the limits of input-focused policy. In contrast, China suggests the potential for rapid improvement when reforms reduce fragmentation and expand primary care capacity. Poland and Turkey highlight how persistent underfunding constrains otherwise coherent institutional models.

Scoring between −10 and −20 points, Group 4 countries face compounded challenges. Russia’s mixed model combines Soviet-era Semashko elements with modern insurance, creating administrative fragmentation. Although insurance is mandatory, regional disparities and low life expectancy- especially among men- drag down national performance. Alcohol abuse, cardiovascular disease, and homicide contribute to high premature mortality (12, 39). Mexico and Brazil have adopted universal or near-universal systems. Brazil’s Unified Health System (Sistema Único de Saúde- SUS) is constitutionally mandated and offers free services nationwide. Mexico employs a segmented insurance model with large uninsured populations relying on public clinics. Both face issues with quality, physician shortages, and urban–rural inequality. High crime rates and chronic underfunding limit system resilience (40).

The challenges in Group 4 systems are amplified by broader socioeconomic instability. High homicide rates in Brazil and Mexico, as well as behavioural risk factors in Russia, increase premature mortality independent of healthcare access. Decentralised governance- particularly in Brazil and Mexico- creates unequal distribution of resources, limiting the impact of universal coverage policies. These findings align with research showing that middle-income health systems remain vulnerable without sustained investment in workforce capacity, surveillance, and chronic disease management.

With z-scores below −45, Group 5 countries reflect the lowest levels of healthcare effectiveness. Despite structural differences, they share limited GDP per capita, high disease burden, and underinvestment. India’s system heavily favours private provision. While public programs like Ayushman Bharat offer limited support for low-income groups, most citizens pay out of pocket, and gaps in diagnostics and specialist services exacerbate inequality (41, 42). Indonesia offers government-subsidised care, but quality and availability vary widely; long wait times and low specialist density hinder timely treatment (43). South Africa’s public system is overburdened, while private insurance is unaffordable for many; the country struggles with dual epidemics of HIV/AIDS and tuberculosis, contributing to elevated mortality (44).

Group 5 countries also face demographic pressures- rapid population growth in India and Indonesia, for example- that strain limited health infrastructure. High out-of-pocket spending and insufficient public investment perpetuate inequities in access and outcomes, particularly in rural areas. Persistent infectious disease burdens divert resources from chronic disease management, further limiting improvements in life expectancy.

The United States represents the clearest example of structural inefficiency within the sample. Despite unparalleled health expenditure, the system struggles with administrative fragmentation, weak price regulation, and incentives that prioritise treatment volume over population outcomes. The absence of a universal baseline benefit package leads to inconsistent access, while employer-linked coverage and means-tested public programmes create discontinuities in insurance status over the life course. These structural features limit the capacity of the system to deliver equitable and cost-effective care.

Compared with Group 1 and Group 2 countries, the U. S. lacks three defining pillars of high-performing systems:

1. A unified regulatory framework for pricing and benefit design.
2. A universal service entitlement ensuring equitable access.
3. Cost-sharing calibrated to encourage prevention rather than defer care.

The absence of these elements contributes to higher administrative costs, lower utilisation of preventive services, and wide socioeconomic disparities in outcomes.

Notably, the United States ranked among the lowest-performing developed countries in the sample, despite having the world’s highest per capita healthcare expenditure. This disconnect highlights inefficiencies that are unlikely to be resolved through higher spending alone. Despite multiple reform efforts, coverage fragmentation and high administrative costs continue to undermine performance (3, 15, 17, 45).

These policy implications should be interpreted as exploratory observations derived from the comparative analysis rather than definitive prescriptions for healthcare reform.

Given political polarisation, a rapid shift to fully publicly funded care seems unlikely in the near term. Moderate reforms inspired by successful models abroad may therefore be more realistic. One example is Germany’s “Krankenkasse” system- a pluralistic, decentralised network of competing health insurance funds (public and private, for-profit and non-profit) with standardised coverage and monthly contributions (7). A similar approach in the U. S. could allow employers or individuals to join regulated health funds with transparent pricing and negotiated service rates. Premiums could remain broadly comparable to current levels, while co-pays and deductibles could be reduced depending on fund design. This model preserves choice and competition while improving equity and reducing administrative burden.

To strengthen prevention incentives, contributions could be linked to preventive actions- such as regular screenings or participation in wellness programs - using behavioural incentives to reduce long-term costs. Countries with robust prevention and early detection suggest that such approaches can strengthen population health while easing fiscal pressure (46). In parallel, broader determinants of health- especially diet and nutrition- should be addressed. High consumption of ultra-processed foods and excess sugar contributes to obesity, diabetes, and cardiovascular disease, increasing system burden (47). Strengthening nutrition education, clearer labelling, and incentives for healthier choices could support cost containment and outcome improvement.

Taken together, these findings imply that the United States would benefit from reforms that prioritise coherence, equity, and preventive incentives rather than additional spending alone. A regulated multi-payer model- anchored in standardised benefits, transparent pricing, and reduced administrative variation- appears to offer a politically feasible pathway. Comparative evidence from Group 1 and Group 2 suggests that reform efforts should emphasise stronger primary care, prevention-oriented incentives, and pricing regulation to reduce unnecessary variation, preserving choice while improving national health performance.

## Limitations

This study has several limitations. First, the analysis relies on publicly available data, primarily from the WHO Global Health Observatory and OECD sources. While these databases are authoritative, some indicators may be affected by inconsistencies in national reporting standards or time lags. Second, the z-score normalisation method enables cross-country comparison but may obscure context-specific nuances, such as regional disparities or cultural factors influencing healthcare access and utilisation.

Third, the selection of 34 indicators, though guided by established frameworks, inevitably involved subjective judgment in determining relevance and data availability. Other valid indicators—such as patient satisfaction, out-of-pocket costs, or care continuity- were excluded due to insufficient data across all countries. Additionally, the analysis does not account for within-country variation, particularly in large or federal nations such as the United States, Brazil, or India, where substantial regional disparities exist in healthcare access, infrastructure, and population health outcomes. National averages may therefore mask important subnational differences in system performance. Finally, while the study proposes a reform model for the United States, further empirical modelling would be required to assess feasibility, political acceptability, and cost-effectiveness under real-world conditions.

## Conclusion

This cross-national analysis demonstrates that healthcare system performance is shaped by a combination of economic capacity, institutional design, and long-term investment in public health infrastructure. While higher national income generally facilitates broader access and better outcomes, the study shows that governance arrangements, regulatory coherence, and preventive care orientation are equally decisive. Systems that achieve the most favourable results tend to exhibit a balanced mix of public financing, structured cost-sharing, transparent pricing, and strong primary care capacity. These structural traits create resilience, reduce inefficiency, and ensure more equitable distribution of care.

The effectiveness of healthcare systems in G20 countries is closely tied to economic development, but financial resources alone are insufficient to guarantee high performance. The countries in the highest-performing group share several institutional characteristics, including regulated insurance markets, structured cost-sharing mechanisms, and strong primary care systems. For developing and transitional economies, a combination of gradual economic growth, investment in health infrastructure and workforce development, and the adoption of structured cost-sharing models offers a viable path toward long-term improvement in health system performance. These findings highlight the importance of aligning economic policy with healthcare system design, and of adopting adaptable, evidence-based frameworks that reflect both local context and global best practices. Comparative analysis, such as the one presented in this study, can serve as a strategic tool for policy innovation, particularly in countries facing resource constraints or seeking to reform underperforming systems. This is particularly relevant as countries seek to strengthen universal health coverage amid rising fiscal constraints, as emphasised by McKee et al. (7).

Ultimately, the study underscores that no single model guarantees success; instead, the most effective systems integrate elements of financing, governance, and prevention into a coherent whole. Policymakers- especially in countries grappling with fragmentation or persistent inequities-may benefit from adopting hybrid approaches that combine choice with solidarity and market incentives with strong regulatory safeguards. As global demographic pressures and the burden of chronic disease continue to rise, the findings highlight the importance of long-term institutional planning, investments in public health capacity, and strategies that prioritise prevention and early detection. These insights offer avenues for reform not only within the G20 but also in emerging economies seeking sustainable, equitable pathways to stronger health system performance.

## Data Availability

The original contributions presented in the study are included in the article/Supplementary material, further inquiries can be directed to the corresponding author.
